# Determination of Mechanical Properties of Blind Rivet Joints Using Numerical Simulations and Experimental Testing

**DOI:** 10.3390/ma18020229

**Published:** 2025-01-07

**Authors:** Martin Beber, Martin Stejskal, Frantisek Sedlacek

**Affiliations:** Faculty of Mechanical Engineering, University of West Bohemia, Univerzitni 2732/8, 301 00 Pilsen, Czech Republic

**Keywords:** rivet joints, blind rivets, tensile performance, FEA, experimental testing

## Abstract

This study explores the tensile performance of blind rivet joints in galvanized steel sheets, focusing on their behavior under shear and normal load conditions. Blind rivets are frequently used in structural applications due to their ease of installation and ability to be applied from one side, making them highly effective in industries like aerospace and automotive. Two types of DIN 7337—4.8 × 8 blind rivets—galvanized steel St/St and stainless steel A2/A2—paired with galvanized steel sheets DX51D + Z275, were experimentally tested to assess how their material properties affect their joint strength, deformation patterns, and failure modes. Single-lap shear, double-lap shear, and pure normal load tests were conducted in multiple configurations to evaluate joint performance under varying loading conditions, simulating real-world stresses. Using custom-built equipment, controlled forces were applied perpendicular to the rivet joints to replicate practical loading conditions. The results revealed distinct differences in the load-bearing capacities of the two materials, offering valuable insights for applications where corrosion resistance and structural integrity are critical. Finite element analysis (FEA) was then used to simulate the behavior of the joints, with the results validated against experimental data. To enhance the reliability of numerical simulations in optimizing the design of rivet joints, a methodology was proposed to calibrate non-linear FEA models to experimental results, and a substantial agreement of 92.53% was achieved via optimization in ANSYS OptiSLang. This research contributes to our broader understanding of riveted connections, providing practical recommendations for assessing the performance of such joints in various engineering fields.

## 1. Introduction

Rivet joints are fundamental components in various engineering and structural applications that provide a reliable means of fastening materials, especially when welding or adhesive bonding is not feasible. Their use is prevalent in steel structures, automotive bodies, and aerospace assemblies, where joint integrity is crucial to overall structural performance. Blind rivets are widely used due to their ease of installation, cost-effectiveness, and ability to form strong joints with access from only one side, a feature that sets them apart from newer methods like clinching and self-piercing rivets, which also enable the joining of various materials [1,2,3].

Structural simulations play a vital role in understanding and enhancing the performance of rivet joints. Finite element analysis (FEA) is a powerful tool for predicting how these joints will behave under various conditions, enabling engineers to optimize designs prior to physical testing. By comparing experimental results with simulation data, computational models can be validated and refined, leading to a more accurate representation of joint behavior. Blind rivet joints have been widely studied, especially in single-lap shear and multi-rivet configurations. Methods like the Rivet Element (RE) model and FEA have proven effective in simulating joint stiffness and behavior while reducing computation time significantly compared to simplified models. Vivio et al. [4] compared Rivet Element performances to a finite element (FE) non-linear model and the experimental data of single-lap shear tests. Mucha [5] studied the single-lap shear tests of various joining systems, including blind rivet and clinching technology.

Experimental and numerical studies on the shear strength of riveted joints typically focus on single-lap shear joints. Quasim et al. [6] studied the influence of parameters such as the hole diameter, length of the rivet, and thickness of the structure on the load-bearing capacity of a single-lap blind rivet joint. Szyca et al. [7] analyzed different parameters of the riveting process and their impact on the fatigue strength of railway connections. Lubas and Bednarz [8] studied the use of a bi-linear material model for the assessment of single-lap shear joints with blind rivets. Lee et al. [9] developed an explicit macroscopic model of a blind rivet to study and predict the influence of various parameters, i.e., adherend thickness, on joint strength, particularly in multi-rivet systems. Liu et al. [10] performed a stress distribution analysis to study the influence of rivet diameter and pitch on the fatigue performance of rivet joints and verified the results with experimental testing.

In thin-walled structures, particularly in the aeronautical industry, riveting deformation has been addressed through local–global finite element method (FEM) approaches that efficiently model lap joints and aircraft panels [11,12,13]. Comparative studies of different rivet types, including blind rivets and solid self-piercing rivets, have established load capacities and joint behavior under shear loads [14,15]. Mucha and Witkowski [15] investigated joint modifications and compared joining techniques, examining their effects on failure modes and destruction processes. FEM is also commonly used to determine the structural effect of material properties, e.g., Li et al. [16] studied methods to enhance the transverse connectivity and structural performance of prestressed concrete box girder bridges through finite element modeling and experimental validation.

This study focuses on the assessment of the tensile behavior of blind rivet joints in galvanized steel sheets through a series of mechanical tests—multiple experimental configurations including pure normal load tests with variable thickness of adherends and single- and double-lap shear tests were used to simulate real-world conditions where tensile and shear stresses are applied, providing critical data. To apply tensile forces directly in the normal direction, i.e., perpendicular to the rivet joint, a custom apparatus was developed, whereas in case of shear loading, standard testing procedures were utilized to apply shear forces through single- and double-lap arrangements. Extensive experimental testing allowed for a comprehensive evaluation of the load-bearing capacity and failure characteristics of rivet joints subjected to normal and shear loads. All tests employed precise hydraulic equipment to apply controlled forces to the joints, while the specimens were assembled using blind rivets made of two distinct materials: galvanized and stainless steel, combined with galvanized steel sheets. This selection of materials offered us a chance to evaluate how the material properties of rivets influence the overall strength and performance of rivet joints. The comparative analysis of these joints under tensile loading conditions revealed differences in their load-bearing capacities, deformation patterns, and failure modes, which are especially relevant for applications requiring corrosion resistance and structural integrity.

While FEM simulations are widely used in such studies, their accuracy in this research was enhanced through direct validation against experimental results from both shear and normal load tests. This approach ensured that the simulations closely matched the tensile behavior of the joints, particularly regarding failure prediction and deformation. By refining the numerical simulations based on experimental data, this study strengthens the reliability of FEM in evaluating the tensile performance of riveted joints, proposing a comprehensive methodology for model calibration to create a robust tool for optimizing designs in thin-walled structures subjected to complex tensile loads.

This study bridges the gap between experimental testing and computational modeling by offering a comprehensive analysis of blind rivet joint performance in galvanized steel sheets. It contributes valuable insights into the design and evaluation of riveted connections in structural engineering.

## 2. Materials and Methods

All galvanized steel sheet specimens were made from the same cold-rolled, hot-dip galvanized mild steel sheet DX51D + Z275 with 275 g/m^2^ of zinc coating, which is commonly used in construction and manufacturing because of its good formability, durability, and corrosion resistance. The yield strength of this material generally varies between 270 and 500 MPa, and its composition can differ depending on the batch—its standard chemical composition and mechanical properties were acquired from the manufacturer and are summarized in Table 1 and Table 2.

**Figure 1:**
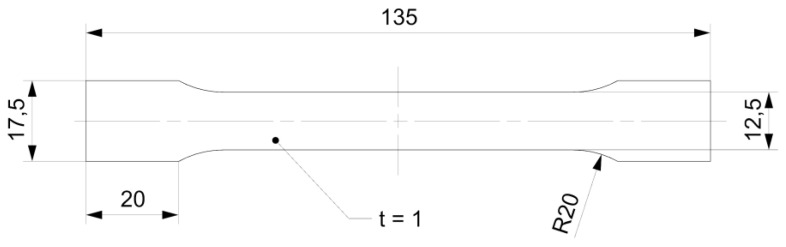
Dimensions of type A galvanized steel sheet specimens according to EN ISO 6892-1.

To identify the exact mechanical properties of the galvanized steel sheets, experimental testing had to be conducted. Therefore, 18 pieces of type A specimen with the dimensions depicted in Figure 1 were manufactured via laser cutting to be used for tensile loading according to EN ISO 6892-1 [17].

**Figure 2:**
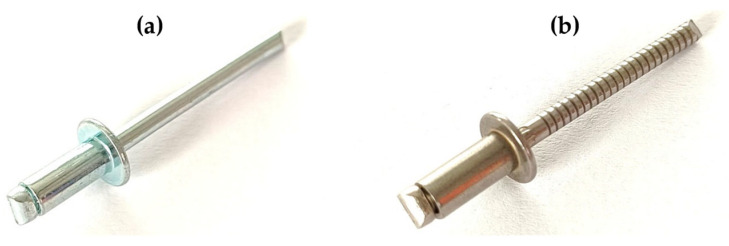
Blind rivets: (**a**) DIN 7337—4.8 × 8 St/St; (**b**) DIN 7337—4.8 × 8 A2/A2.

Subsequently, all riveted joints were made using common steel blind rivets manufactured using two dissimilar materials, both with the same dimensions. Specifically, stainless steel rivets DIN 7337—4.8 × 8 A2/A2 and galvanized carbon steel rivets DIN 7337—4.8 × 8 St/St were utilized, which are shown in Figure 2. The manufacturers were not able to provide the authors with the exact properties of the rivets’ materials, hence the need for extensive experimental testing.

For a blind rivet subjected to pure tensile load, the maximum normal and shear stress $\sigma_{max}$ and $\tau_{max}$ can be calculated using the following equations, which assume the rivet body has a tubular shape:

(1) $$\sigma_{max\;}=\frac{F}{\pi \cdot \left(r_{o}^{2}-r_{i}^{2}\right)},\;\tau_{max}=\frac{V}{2\pi r_{o}t},$$

where F [N] is the normal force, V [N] is the shear force, $r_{o\;(i)}$ [m] is the outer (inner) radius of the rivet, and t [mm] is the wall thickness of the rivet. The exact values of the maximum stresses of DIN 7337—4.8 × 8 rivets are summarized in Table 3.

**Figure 3:**
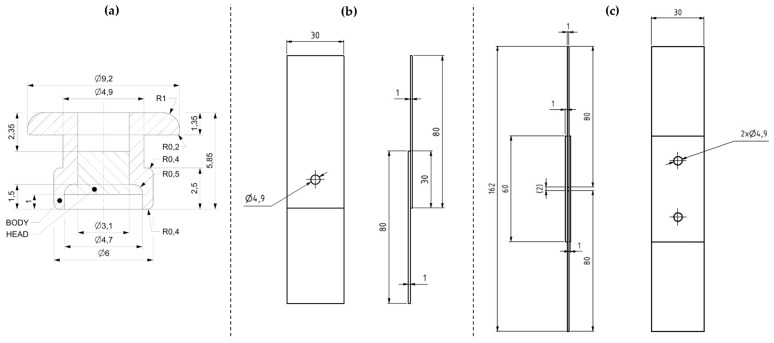
Dimensions of specimens used for experimental testing of riveted joints: (**a**) blind rivet DIN 7337—4.8 × 8 after riveting (**b**) T1 specimens for single-lap shear tensile test; (**c**) T2 specimens used for double-lap shear tensile test.

To identify the exact mechanical properties of the steel blind rivets, a series of experiments was conducted to properly simulate the real-world conditions under which rivet joints are subjected to general, often multi-axial, loading. Therefore, conducting experimental testing with distinct types of loading is crucial for accurately assessing the joint’s behavior under such stresses. In total, fifty-three pieces of T1, T2, and T3 specimens were manufactured to be used for single- and double-lap shear and pure normal tensile loading. The riveted joints were made manually using the Gesipa AccuBird rivet gun (manufactured by SFS Group Germany GmbH Industrial End Markets—GESIPA® in Mörfelden-Walldorf, Germany)—the dimensions of the rivet after riveting are depicted in Figure 3a.

Firstly, specimen T1 depicted in Figure 3b was manufactured to be used in the single-lap shear experiment, where the shear force is continually transformed into tensile force with the displacement of the components. To more precisely assess the deformation and compare it with the computational models, the double-lap specimen T2 depicted in Figure 3c was made to conduct a pure shear loading experiment.

**Figure 4:**
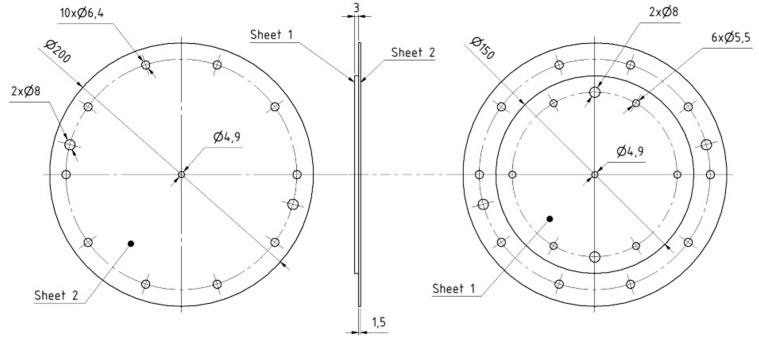
Dimensions of adherends in T3 specimens (t=1.5 mm) used for pure normal tensile test.

**Figure 5:**
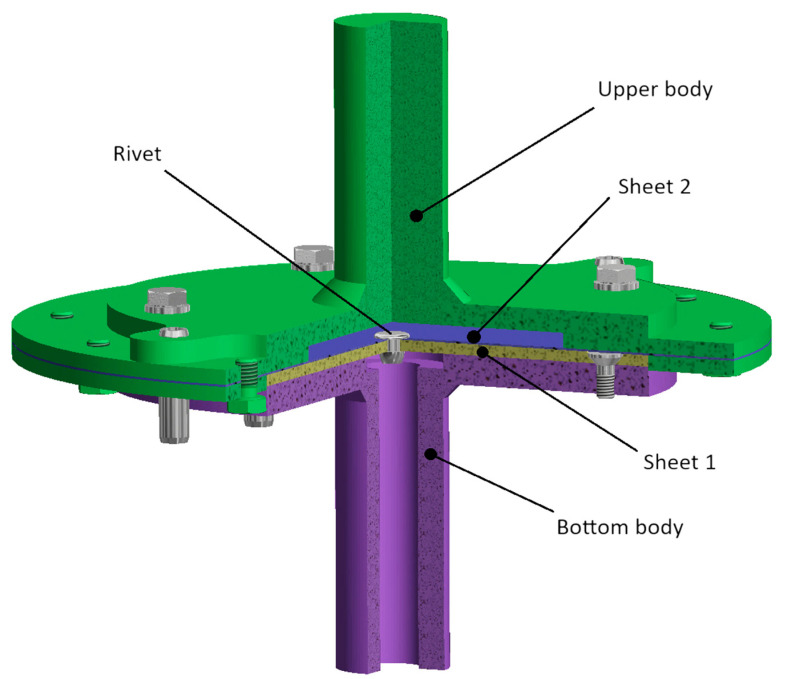
Assembly of testing equipment specially designed for testing pure normal loading of rivets.

Lastly, the T3 specimen depicted in Figure 4 with upper-sheet thickness variations (0.55 mm, 1 mm, 1.5 mm) was prepared for the pure normal loading test. The adherends are a part of a custom piece of equipment designed for the pure normal loading test that can apply tensile forces perpendicular to the plane of the riveted joints, as depicted in Figure 5. In the assembly, there is a Sheet 1 (yellow color) that is bolted to the bottom body (purple color; fixed) and riveted to a Sheet 2 (blue color; top sheet with varying thickness) that is bolted to the upper body (green color; loaded). By applying a tensile load in the normal direction, pure normal loading of the rivet occurs.

## 3. Experimental Testing

### 3.1. Tensile Test of Steel Sheet

**Figure 6:**
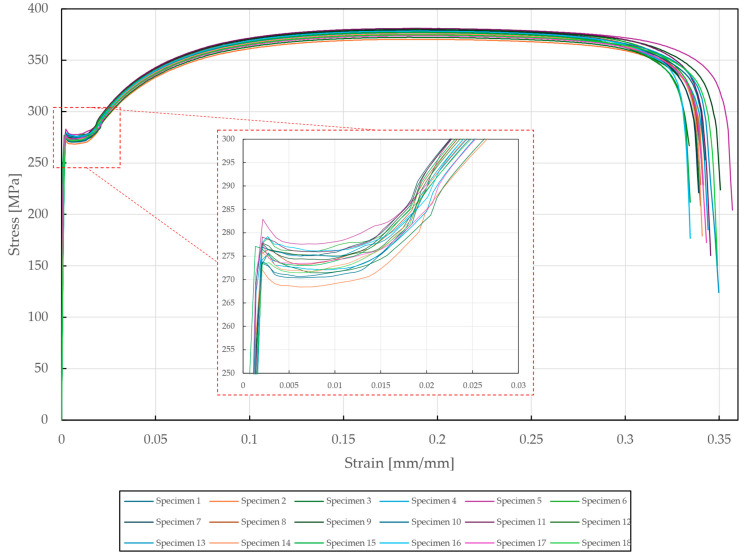
Stress–strain diagram of DX51D + Z275 steel tested according to EN ISO 6892-1.

The tensile load testing of the steel sheet specimens was performed on the Zwick Roell Z250 Tensile testing machine, with the displacement rate fixed at a frequency of 4000 Hz up to the yield strength and then 150 Hz for the rest of the experiment. The results of the tensile tests are depicted in Figure 6. The desired mechanical properties of DX51D + Z275 steel were obtained as median values with absolute deviations—yield strength $R_{eL}=273.2\pm 1.86$ MPa, tensile strength $R_{m}=376.9\pm 2.29$ MPa, and total elongation $A_{80}=34.4\pm 0.22$%. The total difference was found to be approximately 0.64%, confirming the high accuracy of the specimen set. Compared to the values provided by the manufacturer in Table 2, a decrease in yield strength (−13.3%) and total elongation (−13.1%) was found, whereas the difference in tensile strength was negligible (+2.7%).

### 3.2. Tensile Test of Riveted Specimens

**Figure 7:**
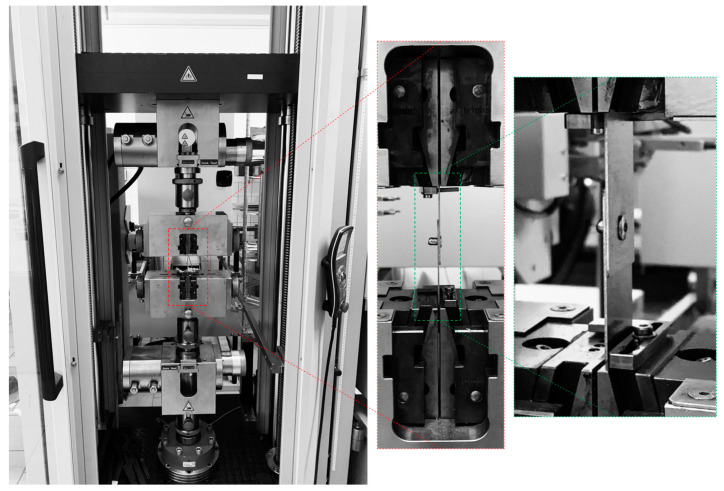
Experimental setup for tensile testing of riveted specimens on Zwick Roell Z250.

**Figure 8:**
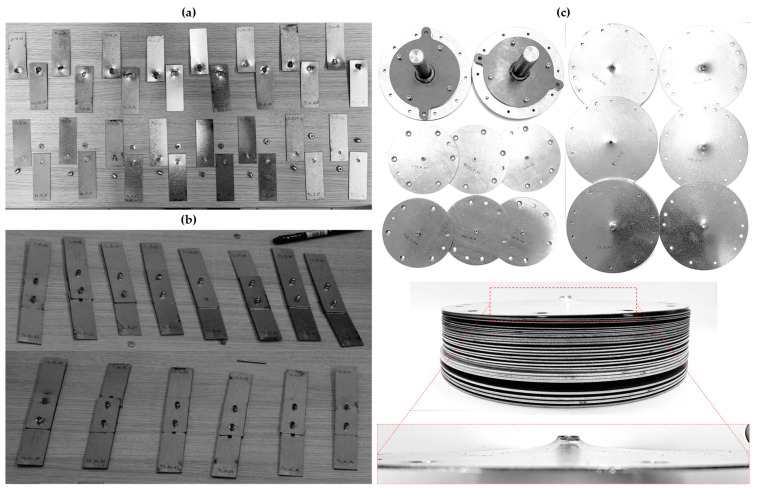
Riveted specimens post experimental tensile testing: (**a**) T1 specimens with A2/A2 (top) and St/St (bottom) rivets subjected to single-lap shear tensile test; (**b**) T2 specimens with A2/A2 (bottom) and St/St (top) rivets subjected to double-lap shear tensile test; (**c**) T3 specimens subjected to pure normal tensile test.

The tensile load testing of the riveted specimens T1, T2, and T3 was performed on the Zwick Roell Z250 Tensile testing machine with the displacement rate fixed at 2 mm/min up to a yield strength and then 5 mm/min for the rest of the experiment. For the specimens T1 and T2, an extensometer was used to obtain the displacement values. The experimental setup and tested specimens are shown in Figure 7 and Figure 8.

**Figure 9:**
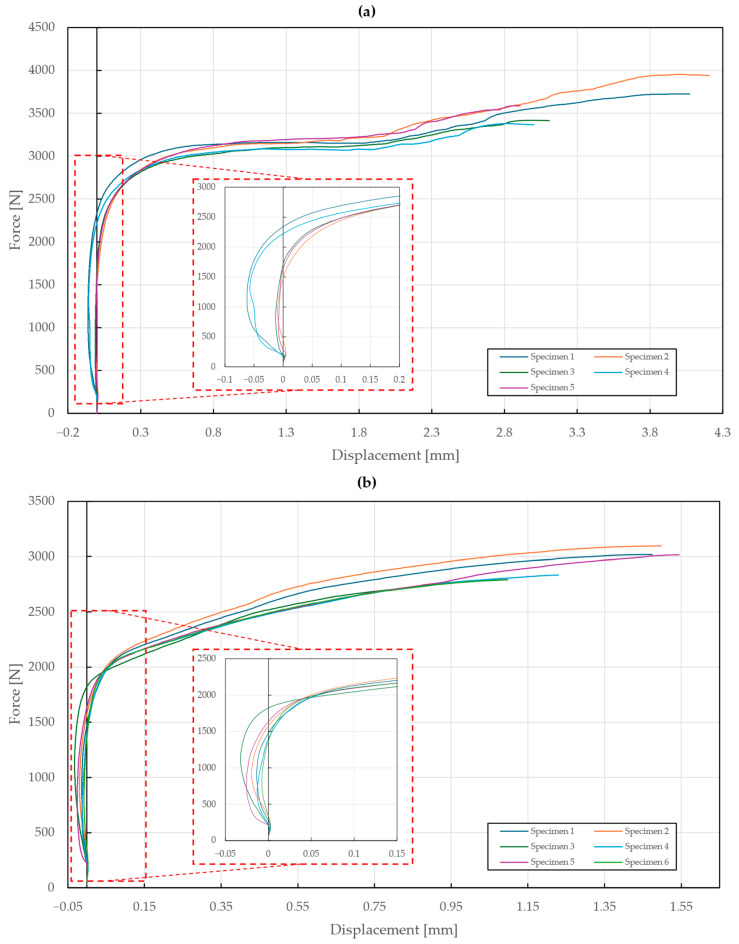
Force–displacement diagram of single-lap shear tensile loading of T1 specimens: (**a**) stainless steel rivets DIN 7337—4.8 × 8 A2/A2; (**b**) galvanized carbon steel rivets DIN 7337—4.8 × 8 St/St.

The results of the tensile testing of specimen T1 are shown in Figure 9. Negative values of displacement were observed in the first part of the tensile diagram, which can be attributed to the seating of the rivet followed by the straightening of the specimens subjected to loading. The yielding point was found to be at around 2500 N (A2/A2 rivet) and 2000 N (St/St rivet). In the case of the A2/A2 rivet, excessive deformations of the riveted steel sheets occurred, which caused the rivet to slip out after reaching a displacement of 3.28±0.37 mm and a force of 3589±174 N, while the rivet itself remained intact. It can be noted that the increase in stiffness before the slippage was caused by additional contact. On the contrary, the testing of specimens with the St/St rivet resulted in a failure of the St/St rivet at a displacement of 1.32±0.20 mm and a force of 2923±114 N, thus confirming that it has the lower load-bearing capacity of the two materials.

**Figure 10:**
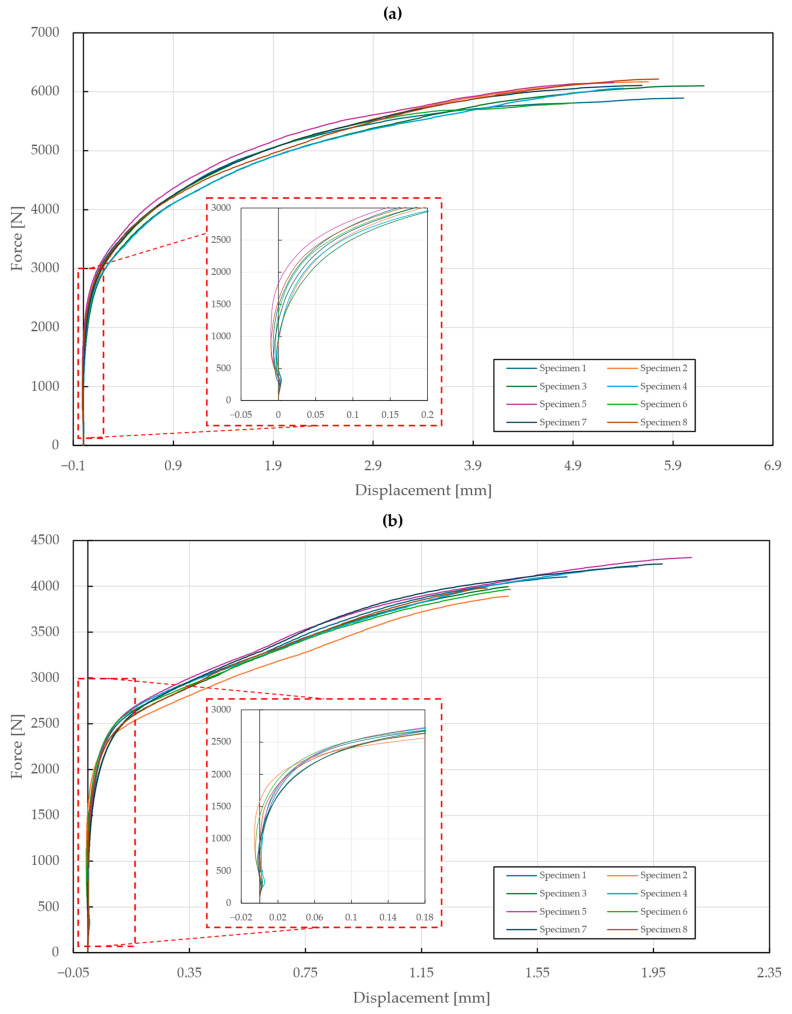
Force–displacement diagram of double-lap shear tensile loading of T2 specimens: (**a**) stainless steel rivets DIN 7337—4.8 × 8 A2/A2; (**b**) galvanized carbon steel rivets DIN 7337—4.8 × 8 St/St.

The results of the tensile testing of T2 specimens are shown in Figure 10. The yielding point was found at around 3000 N (A2/A2 rivets) and 2400 N (St/St rivets). In the case of the A2/A2 rivets, excessive deformations of the riveted steel sheets occurred, which caused one of them to rip apart after reaching a displacement of 5.35±0.35 mm and a force of 6020±129 N, while the rivet remained intact. On the contrary, the testing of specimens with St/St rivets resulted in the failure of one of the rivets at a displacement of 1.67±0.22 mm and force of 4088±125 N. The riveted steel sheets showed almost no signs of plastic deformation.

**Figure 11:**
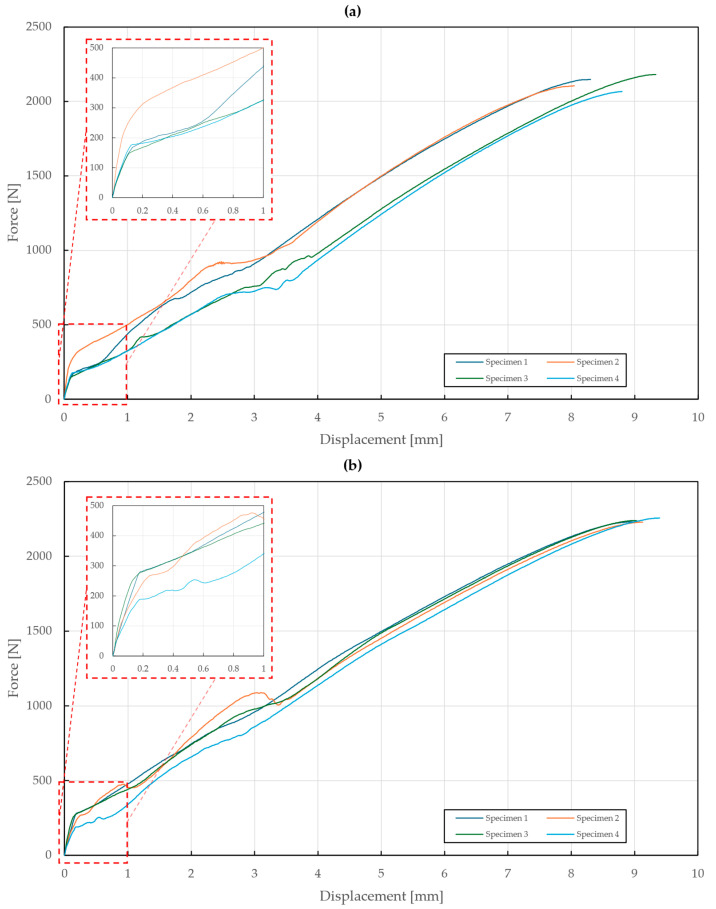
Force–displacement diagram of pure normal tensile loading of T3 specimens (t=0.55 mm): (**a**) stainless steel rivets DIN 7337—4.8 × 8 A2/A2; (**b**) galvanized carbon steel rivets DIN 7337—4.8 × 8 St/St.

**Figure 12:**
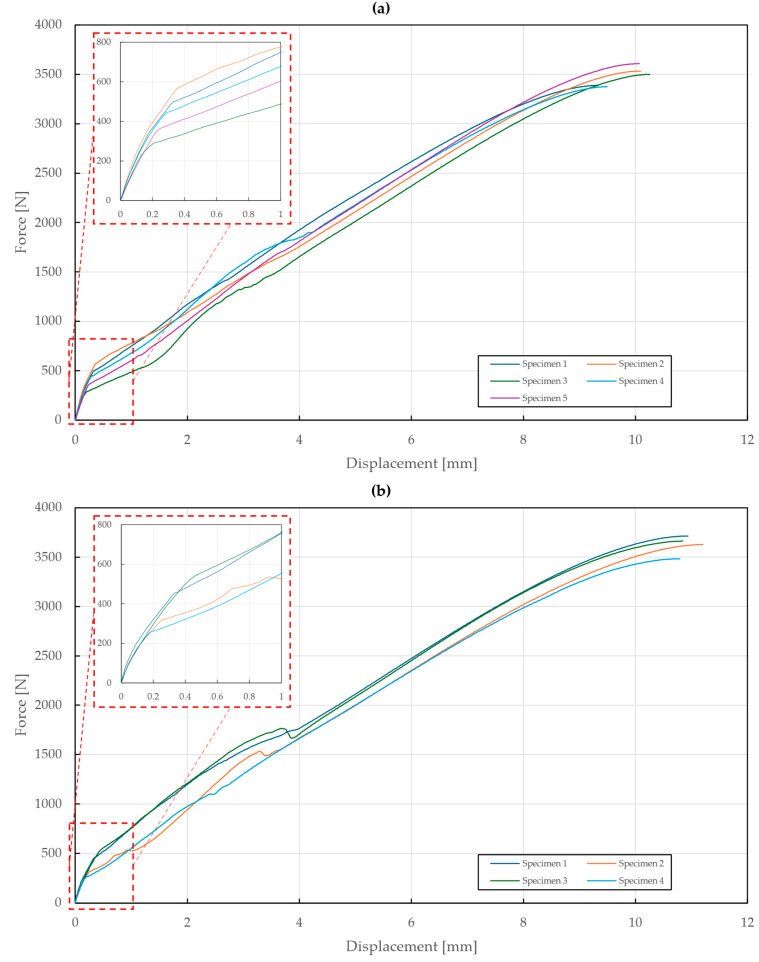
Force–displacement diagram of pure normal tensile loading of T3 specimens (t=1 mm): (**a**) stainless steel rivets DIN 7337—4.8 × 8 A2/A2; (**b**) galvanized carbon steel rivets DIN 7337—4.8 × 8 St/St.

**Figure 13:**
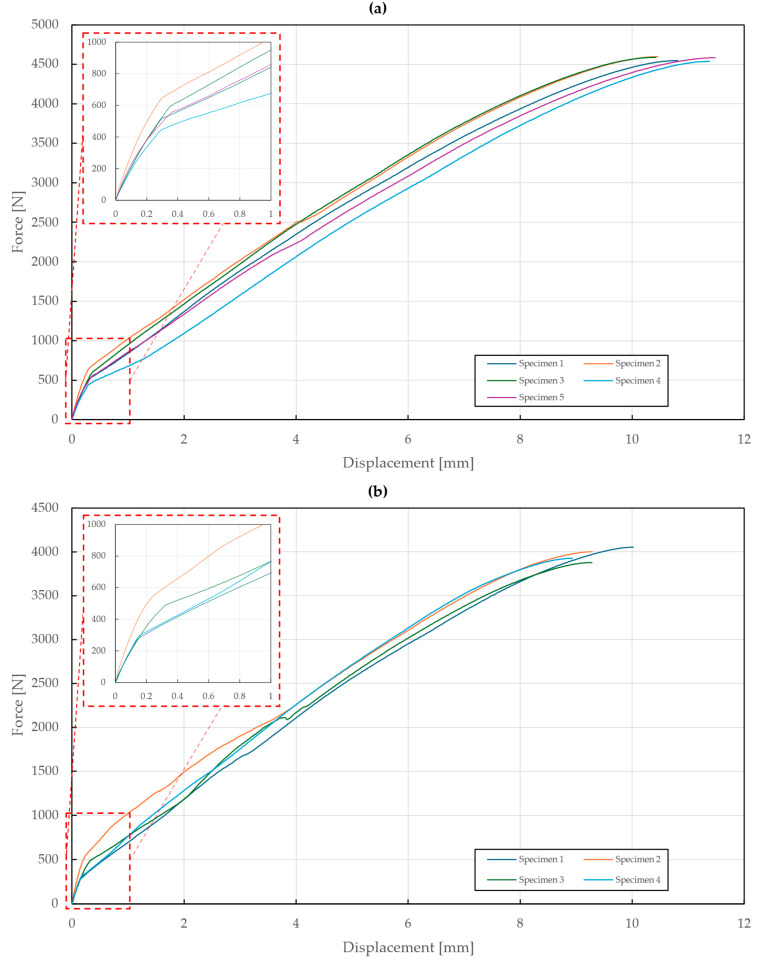
Force–displacement diagram of pure normal tensile loading of T3 specimens (t=1.5 mm): (**a**) stainless steel rivets DIN 7337—4.8 × 8 A2/A2; (**b**) galvanized carbon steel rivets DIN 7337—4.8 × 8 St/St.

In the case of the T3 specimens, rivet variations were supplemented with three variations in the thickness of the upper sheet (0.55 mm, 1 mm, 1.5 mm) and the results are depicted in Figure 11, Figure 12 and Figure 13. The first part of these diagrams is steep as the seating of the components of the specimens and equipment occurred, and the pretension had to be overcome. At a displacement between 3 and 4 mm, some of the diagrams show a force drop due to the separation of the sliding contact of the specimen’s bottom sheet and the equipment’s outer ring.

The results of the specimens with upper sheet thicknesses of 0.55 mm and 1 mm depicted in Figure 11 and Figure 12 showed almost no differences between the A2/A2 and St/St rivets. Moreover, it was observed that all the rivets slipped through the upper steel sheet due to its extensive deformations. For the upper sheet thickness of 0.55 mm, the failure mode was observed at displacements of 8.62±0.45 mm and 9.13±0.13 mm and forces of 2125±40 N and 2239±9 N for the A2/A2 and St/St rivets, respectively.

During the experimental testing of T3 specimens with an upper sheet thickness of 1 mm, both the A2/A2 and St/St rivets slipped through the upper sheet at displacements of 9.85±0.38 mm and 10.94±0.13 mm and forces of 3481±71 N and 3622±66 N.

In the case of a 1.5 mm upper sheet thickness, the St/St rivets failed at a deformation of 9.38±0.27 mm and a force of 3965±63 N, whereas the A2/A2 rivet slipped through the deformed upper sheet at a deformation of 10.91±0.46 mm and a force of 4572±25 N, as shown in Figure 13.

**Figure 14:**
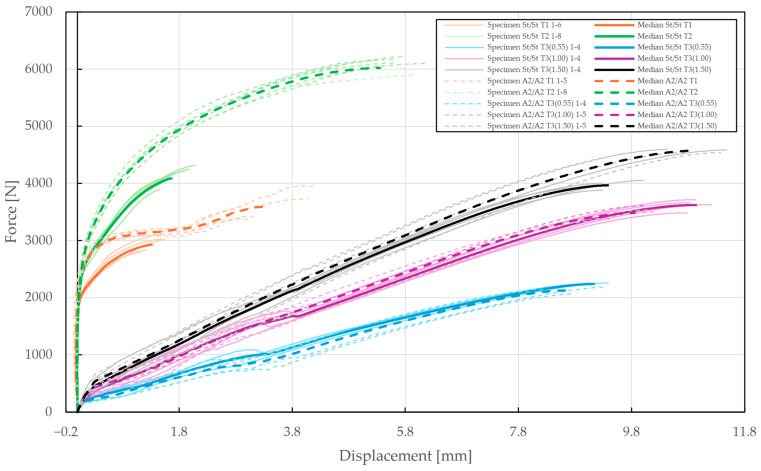
Force–displacement diagram of single-lap shear (T1), double-lap shear (T2), and pure normal loadings (T3) of specimens riveted with DIN 7337—4.8 × 8 A2/A2 and St/St blind rivets.

**Figure 15:**
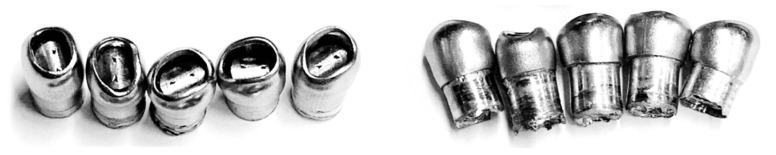
Blind rivets destructed during experimental testing.

To summarize, all results from the experimental testing of the riveted joints, i.e., specimens T1, T2, and T3 and their respective medians, are depicted in overlay in Figure 14. In total, three different failure modes were observed, i.e., the failure or slipping of the rivet or the failure of the steel sheet, which remained consistent across the various specimens and rivet materials. The observed failure mode for each configuration of the riveted specimens is summarized in Table 4, with the slipping of the rivet being the most common (60%). On the contrary, steel sheet failure occurred exclusively during the double-lap shear testing of T2 specimens with an A2/A2 blind rivet. Destructed rivets are depicted in Figure 15.

## 4. Numerical Simulation

### 4.1. General Assumptions and Inputs

To replicate and further study the behavior of various specimens subjected to tensile loading, numerical simulations based on the FEM were employed. A quasi-static implicit solution with perfectly symmetric FE models was assumed. The simulations disregard any imperfections in the specimens, i.e., corrosion, geometric deviations, uneven tightening, welding defects, etc. In terms of boundary conditions, perfectly coaxial loading was assumed and the possibility of specimens slipping in clamps was neglected.

The input CAD geometry used for FE modeling precisely matched the real-world specimens with the dimensions described in Figure 1, Figure 3, and Figure 4. To confirm precise manufacturing, all the specimens were measured using lab-certified measuring devices, revealing a negligible geometric deviation of less than 0.05 mm.

### 4.2. Finite Element Model of Steel Sheet Specimen

**Figure 16:**
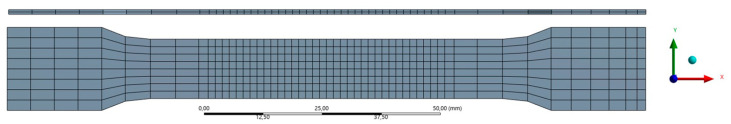
Finite element model of the steel sheet specimens.

The tensile loading of steel sheet specimens was numerically simulated in ANSYS Mechanical 2024R1 FE software. A three-dimensional non-linear FE model consisting of 864 linear Hex8 elements was created and is depicted in Figure 16.

To accurately represent the tensile behavior of the steel sheet, Chaboche kinematic hardening was specified, along with isotropic elasticity. The calibration of material data and the fitting of the numerical simulation were carried out utilizing ANSYS optiSLang 2024R1. For this purpose, One-Click Optimization (OCO) was used to minimize the total difference ${∆}_{c}$ between the numerical and true experimental stress–strain curves σ(ε), with the emphasis on the linear section of the curve via the following user-defined criteria:

(2) $${∆}_{C}=∆+\dot{∆}=\frac{{\left\|\sigma_{S}\left(\varepsilon_{i}\right)-\sigma_{E}(\varepsilon_{i})\right\|}_{2}}{\max\sigma_{E}}+\frac{{\left\|\dot{\sigma_{S}}\left(\varepsilon_{i}\right)-\dot{\sigma_{E}}(\varepsilon_{i})\right\|}_{2}}{\max{\dot{\sigma }}_{E}},$$

where ∆ and $\dot{∆}$ are the Euclidean distances, i.e., absolute difference between the numerical (lower index *S*) and experimental (lower index *E*) stress–strain curves and its first derivative.

### 4.3. Finite Element Model of Riveted Specimens

**Figure 17:**
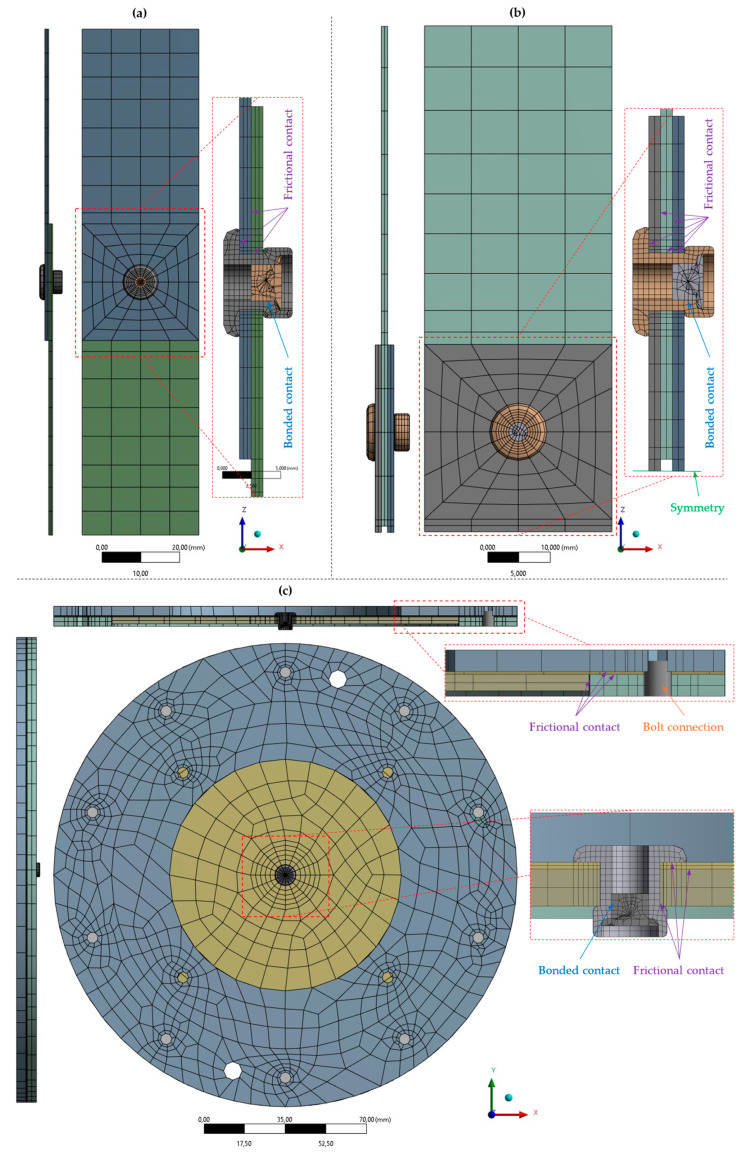
Finite element model of riveted specimens: (**a**) T1 specimen for single-lap shear loading; (**b**) T2 specimen for double-lap shear loading; and (**c**) T3 specimen for pure normal loading of rivet joint.

The shear and normal loading of riveted specimens was conducted in ANSYS Mechanical 2024R1 FE software. Multiple three-dimensional non-linear FE models consisting of mostly linear Hex8 elements were created. The single- and double-lap shear specimens are shown in Figure 17a,b, respectively, whereas the specimens subjected to normal loading, which vary solely in the thickness of their upper sheet (0.55 mm, 1 mm, 1.5 mm), are shown in Figure 17c. The bolt connections were modeled using the beam elements, whereas the interaction between each body was specified as a frictional contact. To improve computational efficiency, reasonable simplifications were made, e.g., symmetry, and to obtain more realistic results, both the bolts and rivets were preloaded, and large deformations were assumed.

Similarly to the steel sheet, Chaboche kinematic hardening and isotropic elasticity were used to describe the shear and tensile behavior of both the A2/A2 and St/St rivets. The calibration of the material data and the fitting of the numerical simulation were carried out using ANSYS optiSLang 2024R1, with the main emphasis on the critical sections of the force–displacement curve F(δ). Based on a mesh sensitivity study, a less precise discretization, i.e., coarser mesh, of the specimens’ FE models was used due to the repetitiveness of fitting non-linear numerical simulations to experimental results.

## 5. Results

### 5.1. Fitting the Numerical Simulation

**Figure 18:**
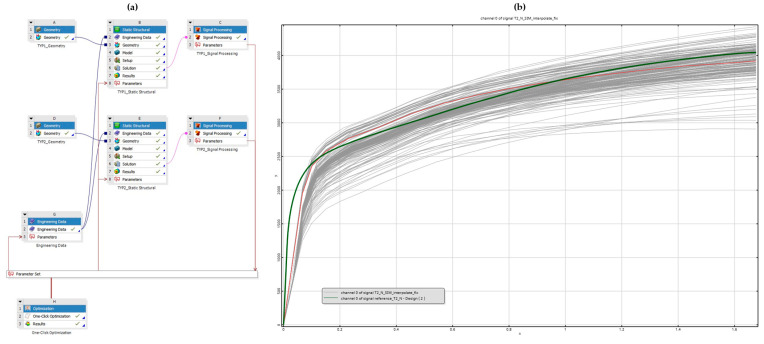
Overview of the numerical simulation’s fitting: (**a**) parametrized FE model with automated post-processing of two parallel simulations; (**b**) model calibration of double-lap shear T2 specimen (green is the reference force–displacement curve from the experiment).

The fitting of numerical simulations consisted of finding the global minimum of the total difference ${∆}_{c}$ between two force–displacement curves and was performed using the optimization capabilities of ANSYS optiSLang 2024R1. For this purpose, fully parametrized non-linear FE models with automated post-processing were created. An overview of the optimization setup and subsequent model calibration is shown in Figure 18.

Firstly, the fitting of the tensile testing of the steel sheet specimens was conducted to provide input data for the numerical simulations of the riveted specimens. Then, the model calibration of rivet joints subjected to shear loading, i.e., specimens T1 and T2, was conducted in a similar setting. The pure normal T3 specimens were omitted from the fitting because the experiments revealed their insensitivity to the rivet material, which helped to reduce the computation time and the complexity of the model’s calibration. Lastly, the results were validated and fine-tuned using a more precise discretization of the FE models.

### 5.2. Model Calibration of Steel Sheet Specimen

**Figure 19:**
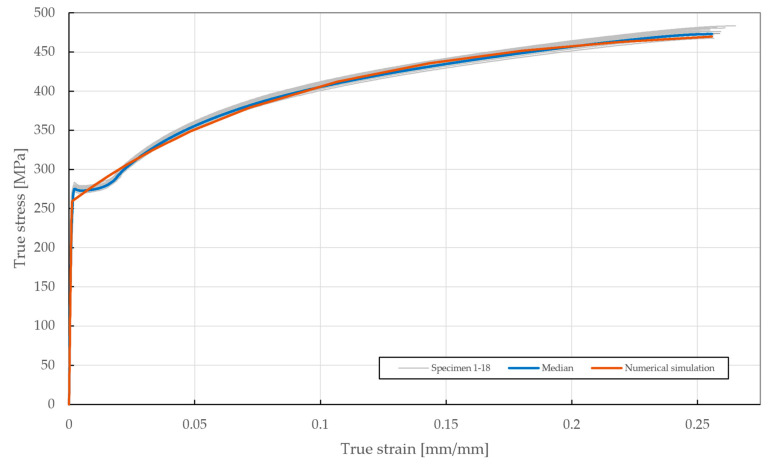
True stress–strain curve of the DX51D + Z275 steel sheet specimen experimentally tested according to EN ISO 6892-1 and the results of the fitted non-linear numerical simulation.

The results from the numerical simulation of the tensile loading of the steel sheet specimens are shown in Figure 19, where a very good agreement (∆≅0.79, $\dot{∆}≅1.33$) with the median of the experimental results can be seen. The best-fitting material parameters of DX51D + Z275 steel are summarized in Table 5. In terms of Chaboche parameters, the material constant $C_{1}$ represents the tangent modulus past the yield strength $R_{e}$, whereas the material constant $\gamma_{1}$ makes the curve past the yield point linear ($\gamma_{1}=0$), convex ($\gamma_{1}<0$), or concave ($\gamma_{1}>0$) depending on its value.

It was found that the absolute difference ∆ was mostly influenced by the yield strength $R_{e}$ (66%) and material constants $\gamma_{1}$ (60%) and $C_{1}$ (39%). On the contrary, the Young’s modulus E was determined to be the most influential parameter (61%) in terms of the difference in the first derivative $\dot{∆}$, despite its negligible effects (3%) on the difference in ∆. Despite its inclusion in the model’s calibration, Poisson’s ratio μ did not affect any of the differences, thus confirming the correctness of the numerical simulation and its fitting to the experimental results of the uniaxial tensile test. To obtain its exact value, additional experiments would have to be conducted, i.e., based on ASTM E132 [18].

### 5.3. Model Calibration of Riveted Specimens

**Figure 20:**
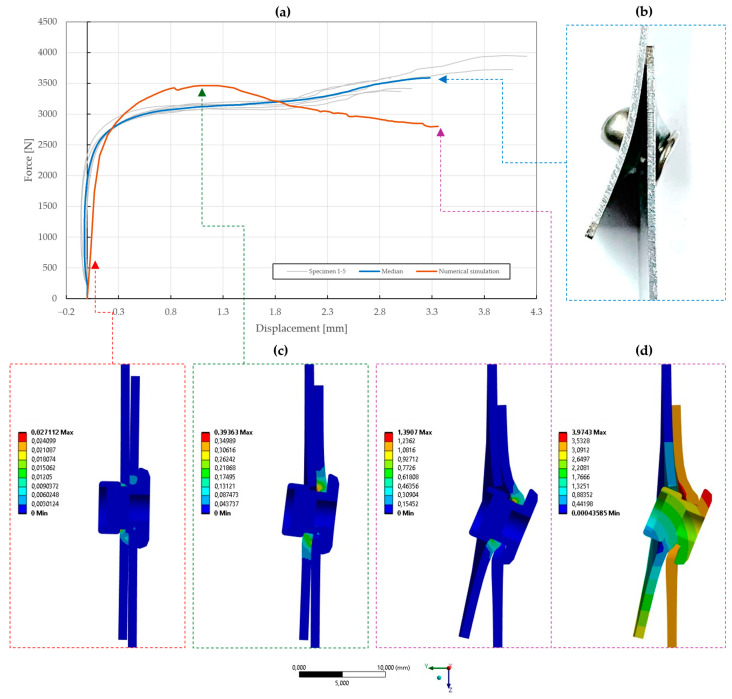
Force–displacement curve of single-lap shear T1 specimen riveted with DIN 7337—4.8 × 8 A2/A2 blind rivet and the results of its fitted non-linear numerical simulation: (**a**) force–displacement curve; (**b**) deformation after experimental tensile testing; (**c**) results of equivalent plastic strain from numerical simulation [mm/mm]; (**d**) results of total deformation from numerical simulation [mm].

**Figure 21:**
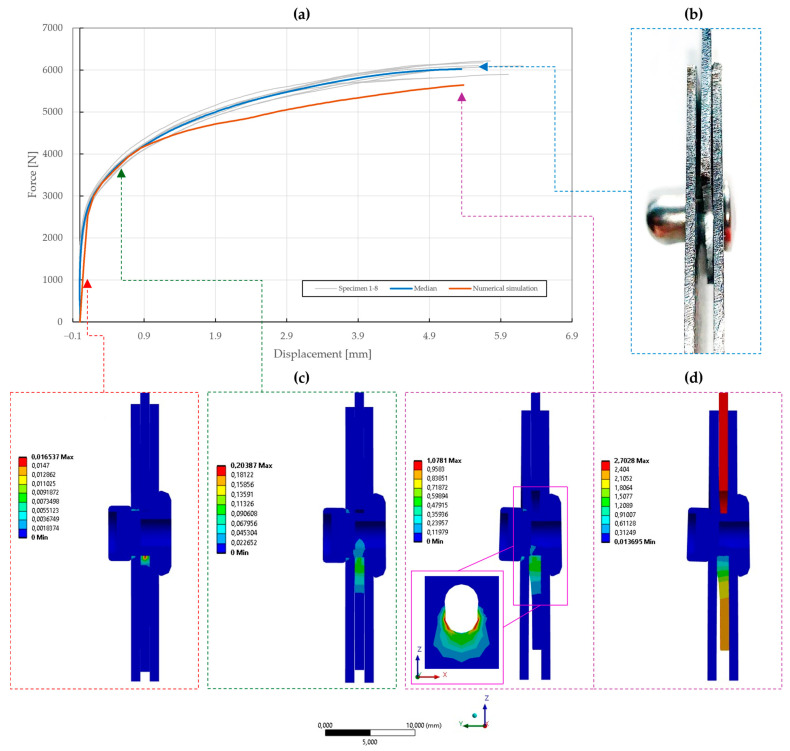
Force–displacement curve of double-lap shear T2 specimen riveted with DIN 7337—4.8 × 8 A2/A2 blind rivet and the results of its fitted non-linear numerical simulation: (**a**) force–displacement curve; (**b**) deformation after experimental tensile testing; (**c**) results of equivalent plastic strain from numerical simulation [mm/mm]; (**d**) results of total deformation from numerical simulation [mm].

**Figure 22:**
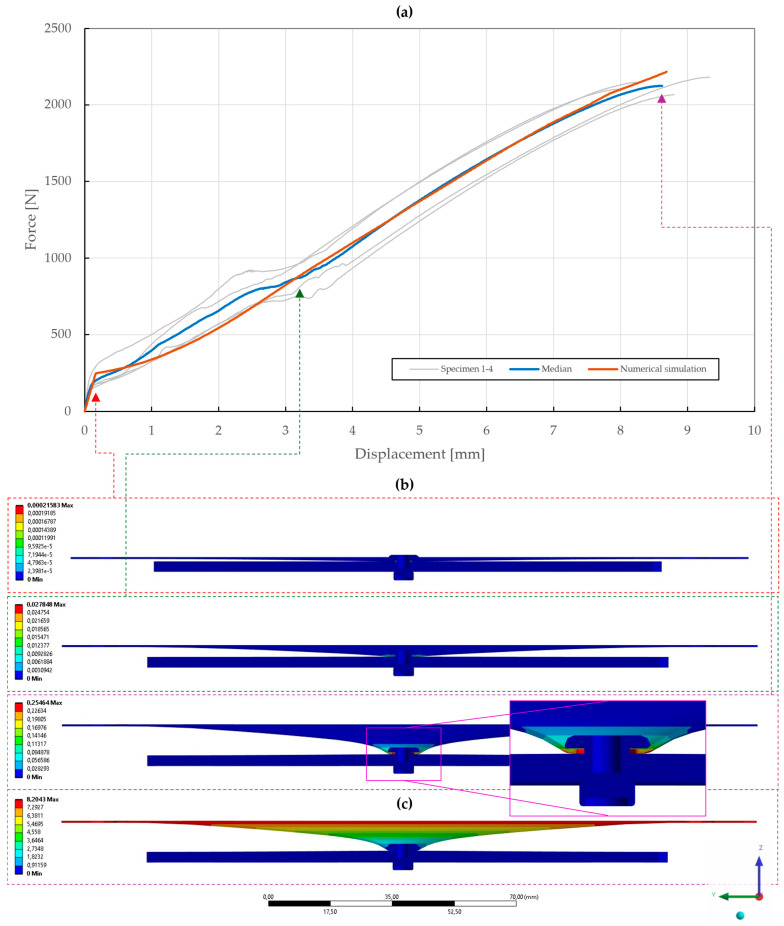
Force–displacement curve of pure normal T3 specimen (t=0.55 mm) riveted with DIN 7337—4.8 × 8 A2/A2 blind rivet and the results of its fitted non-linear numerical simulation: (**a**) force–displacement curve; (**b**) results of equivalent plastic strain from numerical simulation [mm/mm]; (**c**) results of total deformation from numerical simulation [mm].

**Figure 23:**
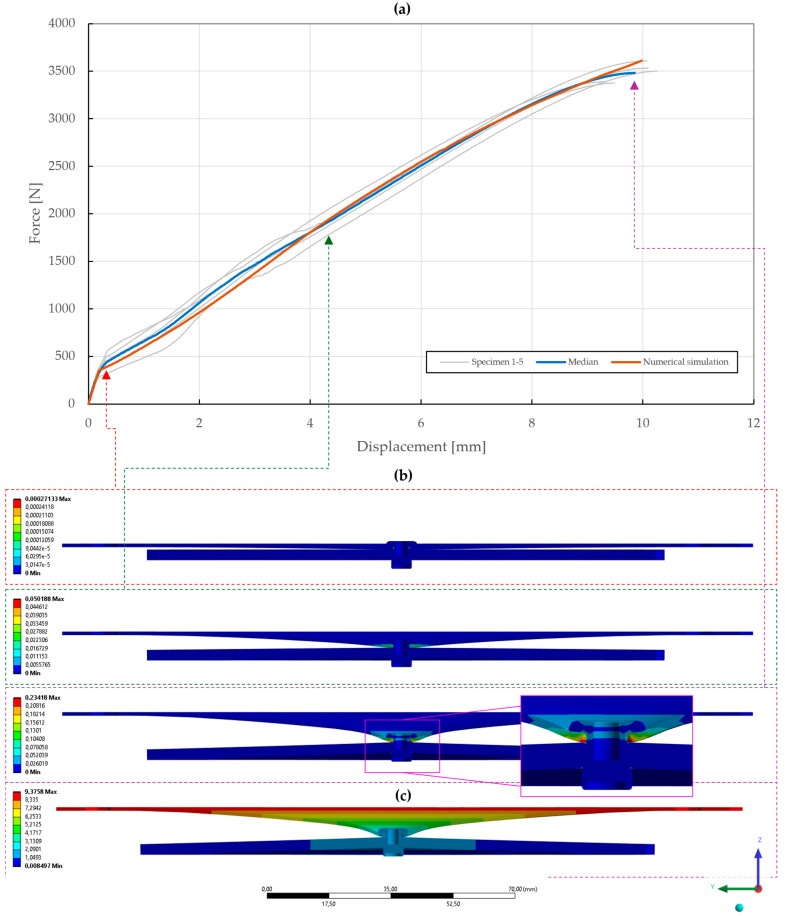
Force–displacement curve of pure normal T3 specimen (t=1.00 mm) riveted with DIN 7337—4.8 × 8 A2/A2 blind rivet and the results of its fitted non-linear numerical simulation: (**a**) force–displacement curve; (**b**) results of equivalent plastic strain from numerical simulation [mm/mm]; (**c**) results of total deformation from numerical simulation [mm].

**Figure 24:**
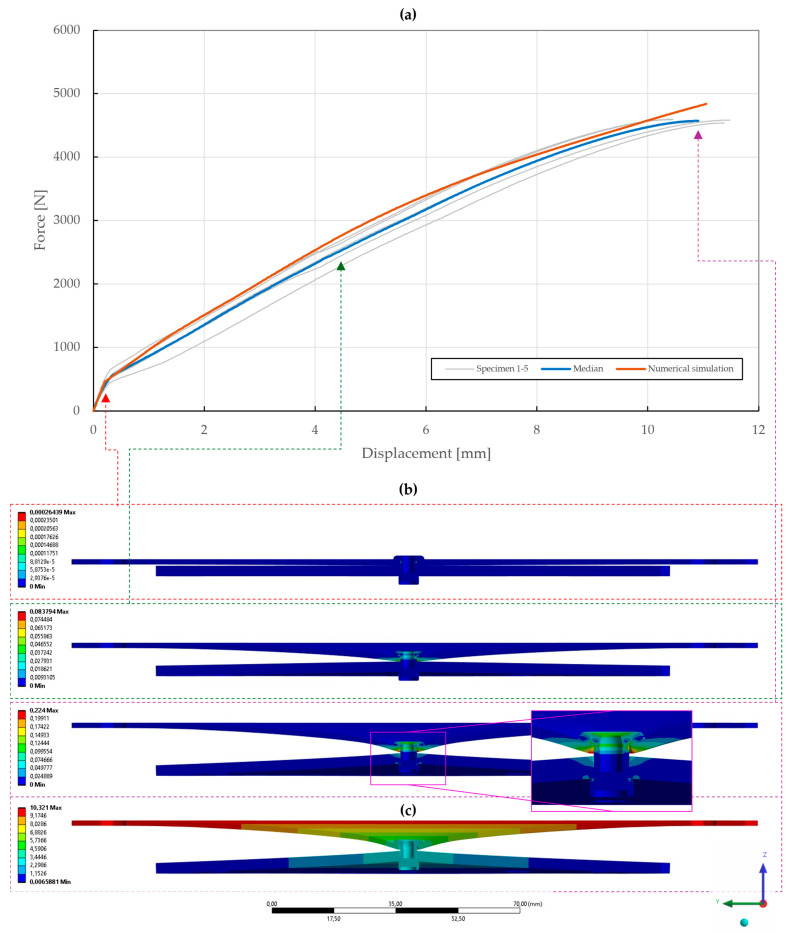
Force–displacement curve of pure normal T3 specimen (t=1.50 mm) riveted with DIN 7337—4.8 × 8 A2/A2 blind rivet and the results of its fitted non-linear numerical simulation: (**a**) force–displacement curve; (**b**) results of equivalent plastic strain from numerical simulation [mm/mm]; (**c**) results of total deformation from numerical simulation [mm].

**Figure 25:**
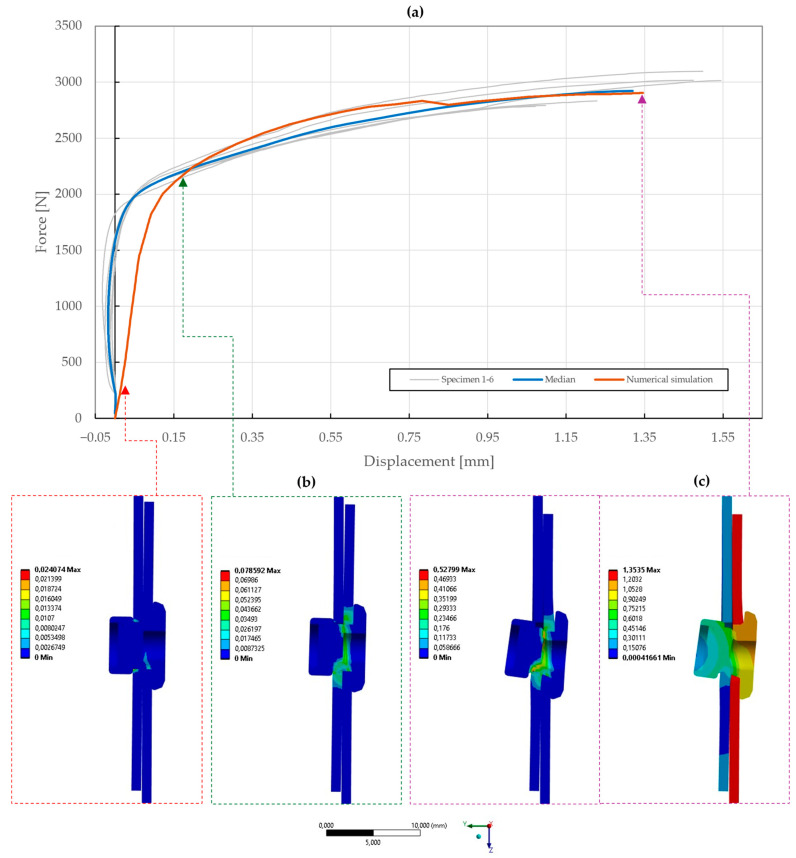
Force–displacement curve of single-lap shear T1 specimen riveted with DIN 7337—4.8 × 8 St/St blind rivet and the results of its fitted non-linear numerical simulation: (**a**) force–displacement curve; (**b**) results of equivalent plastic strain from numerical simulation [mm/mm]; (**c**) results of total deformation from numerical simulation [mm].

**Figure 26:**
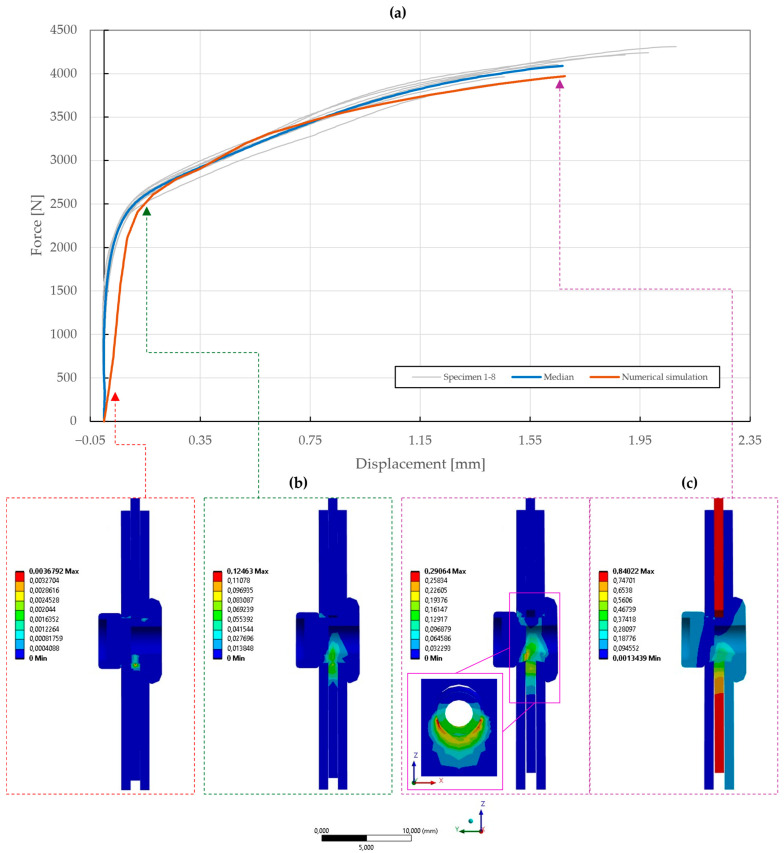
Force–displacement curve of double-lap shear T2 specimen riveted with DIN 7337—4.8 × 8 St/St blind rivet and the results of its fitted non-linear numerical simulation: (**a**) force–displacement curve; (**b**) results of equivalent plastic strain from numerical simulation [mm/mm]; (**c**) results of total deformation from numerical simulation [mm].

**Figure 27:**
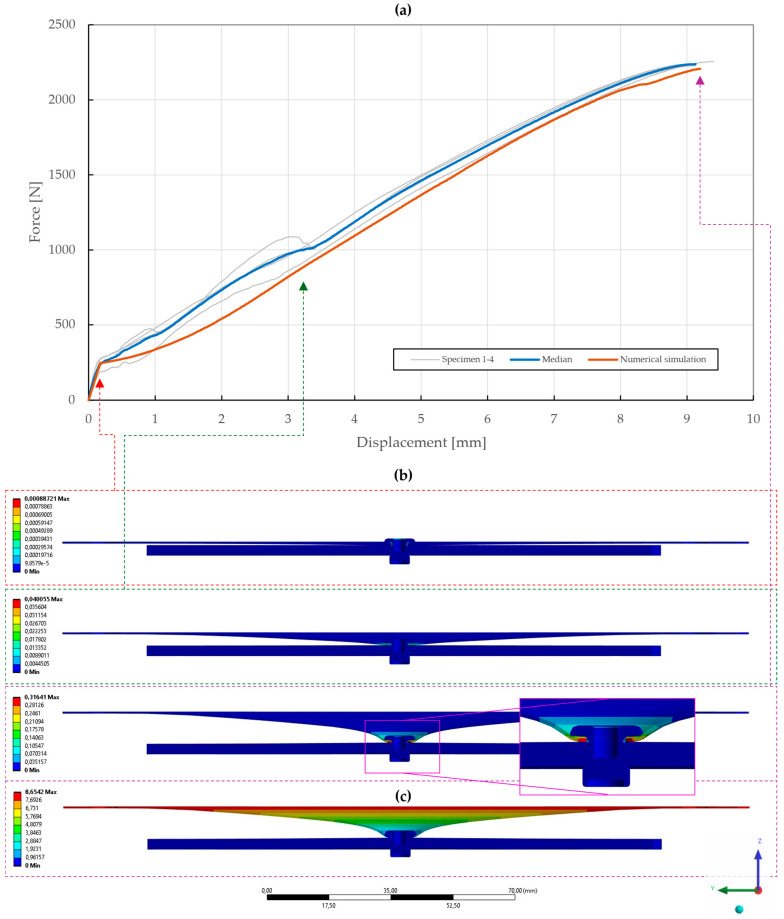
Force–displacement curve of pure normal T3 specimen (t=0.55 mm) riveted with DIN 7337—4.8 × 8 St/St blind rivet and the results of its fitted non-linear numerical simulation: (**a**) force–displacement curve; (**b**) results of equivalent plastic strain from numerical simulation [mm/mm]; (**c**) results of total deformation from numerical simulation [mm].

**Figure 28:**
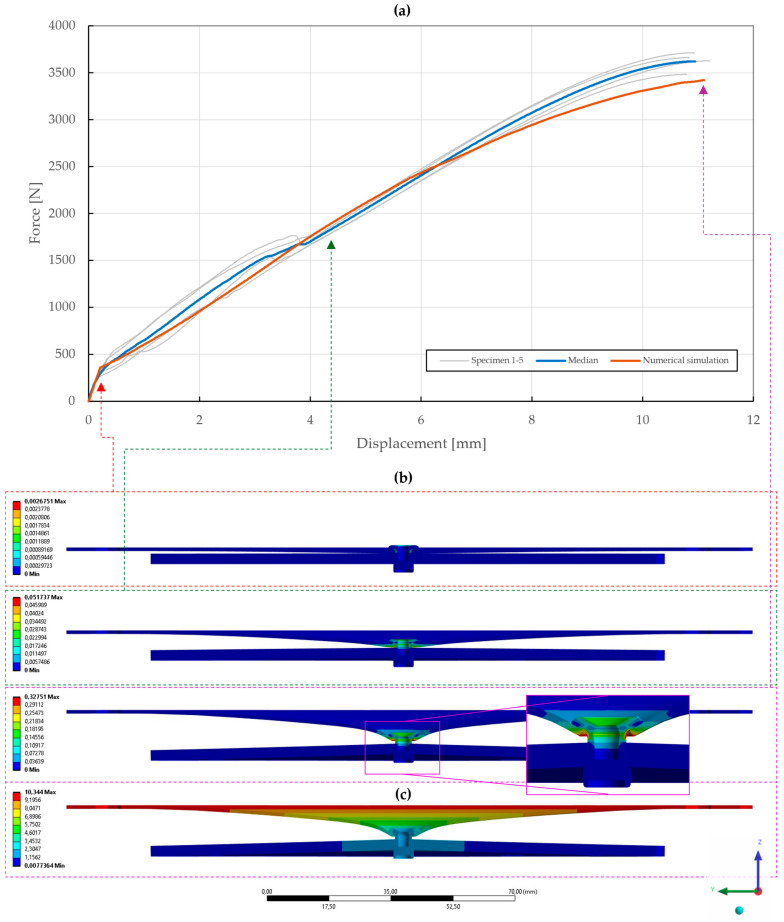
Force–displacement curve of pure normal T3 specimen (t=1.00 mm) riveted with DIN 7337—4.8 × 8 St/St blind rivet and the results of its fitted non-linear numerical simulation: (**a**) force–displacement curve; (**b**) results of equivalent plastic strain from numerical simulation [mm/mm]; (**c**) results of total deformation from numerical simulation [mm].

**Figure 29:**
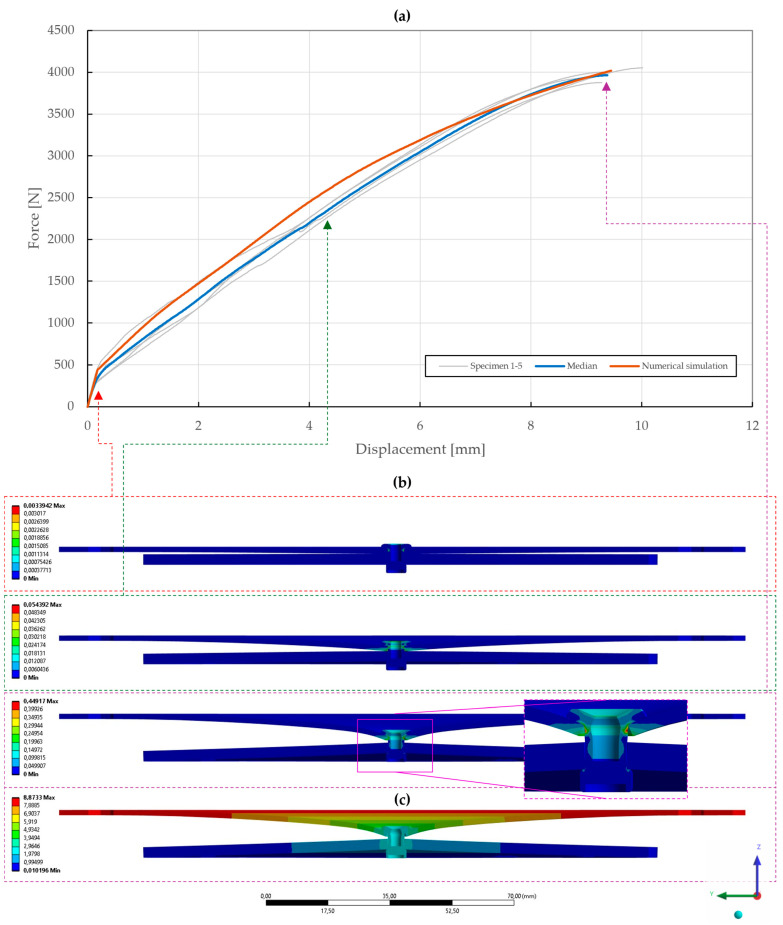
Force–displacement curve of pure normal T3 specimen (t=1.50 mm) riveted with DIN 7337—4.8 × 8 St/St blind rivet and the results of its fitted non-linear numerical simulation: (**a**) force–displacement curve; (**b**) results of equivalent plastic strain from numerical simulation [mm/mm]; (**c**) results of total deformation from numerical simulation [mm].

The results of the model calibration of the riveted specimens are shown in Figure 20, Figure 21, Figure 22, Figure 23, Figure 24, Figure 25, Figure 26, Figure 27, Figure 28 and Figure 29, where a very good agreement with the median of all the experimental results can be seen, thus proving the correctness of the selected fitting methodology. Moreover, both the failure modes and results of the deformations in the fitted numerical simulations closely match the real-world behavior of rivet joints subjected to shear and normal loading. The best-fitting material parameters of DIN 7337—4.8 × 8 blind rivets are summarized in Table 6.

For the stainless-steel blind rivet DIN 7337—4.8 × 8 A2/A2, an absolute difference ∆ of 5.81 and 3.02 was achieved for the force–displacement curves of the single- and double-lap shear specimens T1 and T2, respectively. Further fine-tuning of the numerical simulations was not feasible due to the insufficient stiffness of the adherends, resulting in an increase in compliance of the rivet joint at higher displacements. Despite this, the results of the deformation, depicted in Figure 20b,d and Figure 21b,d, are consistent with the experimental results. A similar trend was also observed for the initiation and further evolution of the failure mode, shown as results of the equivalent plastic strain in Figure 20c and Figure 21c. From a statistical standpoint, the coefficient of importance to the difference ∆ in specimens T1 and T2 was found to be the greatest for yield strength $R_{e}$ (80% and 68%), material constants $C_{1}$ (13% and 35%) and $\gamma_{1}$ (2% and 8%), and rivet pretension (4% and 1%). Due to geometric non-linearities, i.e., frictional contacts and the sliding of the adhered surfaces, the coefficient of friction was also found to be significant.

With the obtained material parameters, the numerical simulation of pure normal loaded T3 specimens (t=0.55,1.00,1.50 mm) was fine-tuned and a substantial agreement (∆=0.71,0.46,1.04; $\dot{∆}=1.64,1.44,1.48$) was achieved in comparison to the experimental data, as shown in Figure 22, Figure 23 and Figure 24. Here, it was found that the contact settings, i.e., maximum allowed penetration, along with pretension of both the bolts and rivet impact the stiffness of a rivet joint loaded in the normal direction. When comparing the evolutions of the equivalent plastic strain, it also appears that the increase in thickness, i.e., stiffness, of the upper steel plate translates to an increase in damage to the rivet, as the deformation energy is not spent on the deflection of the stiffer adherends. Therefore, a slight inflection point in the force–displacement curve can be observed when the rivet starts to plasticize.

The numerical simulation of the galvanized carbon steel blind rivet DIN 7337—4.8 × 8 St/St was calibrated in an equivalent manner, resulting in a difference ∆ of 2.21 and 2.56 for the specimens T1 and T2 subjected to shear loading. Compared to the A2/A2 rivet, a lower load-bearing capacity and different failure modes of the St/St rivet were observed, conforming to the results of the tensile experiments, as depicted in Figure 25 and Figure 26. The coefficient of importance to the results of specimens T1 and T2 was found to be more diversely distributed. Specifically, the yield strength $R_{e}$ (11% and 49%), material constants $C_{1}$ (17% and 29%) and $\gamma_{1}$ (23% and 7%), and Young’s modulus E (5% and 9%) were found to be the most influential parameters.

Finally, the pure normal T3 specimens (t=0.55,1.00,1.50 mm) with St/St rivets were also fitted with a great agreement (∆=1.36,1.06,1.14; $\dot{∆}=1.31,1.64,1.44$). The results are presented in Figure 27, Figure 28 and Figure 29. It was observed that when the deformation energy is spent on the plasticization of the rivet, the force–displacement curve becomes concave past the initial linear region.

## 6. Discussion

To study the behavior of a rivet joint subjected to various loading conditions, single- and double-lap shear configurations were used for the shear loading, while a custom-made specimen was designed for pure normal loading. For this purpose, blind rivets DIN 7337—4.8 × 8 made of two distinct materials, i.e., stainless steel A2/A2 and galvanized carbon steel St/St, were used. Experimental tensile tests of the galvanized steel sheets DX51D + Z275 were conducted in accordance with the EN ISO 6892-1 standard. Moreover, this extensive experimental testing was supported by non-linear numerical simulations based on the FE approach.

In the single-lap shear configuration (T1 specimens), shear forces induced additional tensile stress due to specimen displacement. The A2/A2 rivets exhibited a higher load-bearing capacity, yielding at approximately 2500 N, and failing due to rivet slippage at 3589±174 N. The St/St rivets yielded at around 2000 N and failed earlier at 2923±114 N due to rivet fracture. The bending effects inherent in single-lap joints reduced the overall strength of the joint compared to the double-lap shear configuration.

The double-lap shear setup (T2 specimens) provided a more uniform shear load, eliminating the bending which occurs in single-lap joints. The A2/A2 rivets withstood forces of approximately 6020±129 N, with failure occurring in the steel sheets rather than the rivets—indicating that the rivet strength exceeded that of the sheet material. The St/St rivets failed at lower loads (4088±125 N) due to rivet fracture, with minimal sheet deformation. The double-lap configuration enhanced joint strength and demonstrated the superior performance of stainless-steel rivets under pure shear loads.

In the case of a normal load (T3 specimens), where tensile force was applied perpendicular to the joint plane, both rivet types showed a similar performance with thinner upper sheets (0.55 mm and 1 mm), primarily failing due to the rivet slipping through the deformed adherends. With a thicker upper sheet (1.5 mm), the A2/A2 rivets outperformed the St/St rivets, sustaining higher loads (4572±25 N vs. 3965 ± 63 N) and greater deformations before the failure. In this configuration, the A2/A2 rivet slipped through, whereas the St/St rivet was destroyed. This suggests that increased sheet thickness enhances the stiffness of the joint, resulting in a higher load-bearing capacity while eliminating the possibility of slippage. However, it can be assumed that with a further increase in the thickness of the rivetted sheets, the accumulated deformation energy causes the rivet to deform past its yield point, with failure occurring at lower displacements. Moreover, stainless steel rivets offer slightly better performance under tensile stress when compared to galvanized carbon steel rivets.

The numerical simulation was fitted to accurately represent the real-world behavior of the rivet joints subjected to shear and normal loading. Firstly, the material parameters of the adherends were identified and calibrated. Then, the material parameters of rivets subjected to shear loading in single- and double-lap configurations (T1 and T2 specimens) were optimized. Subsequently, the numerical simulation of pure normal tensile loading (T3 specimens) was fine-tuned. And, finally, the FE models were refined to allow for more accurate result distributions.

**Figure 30:**
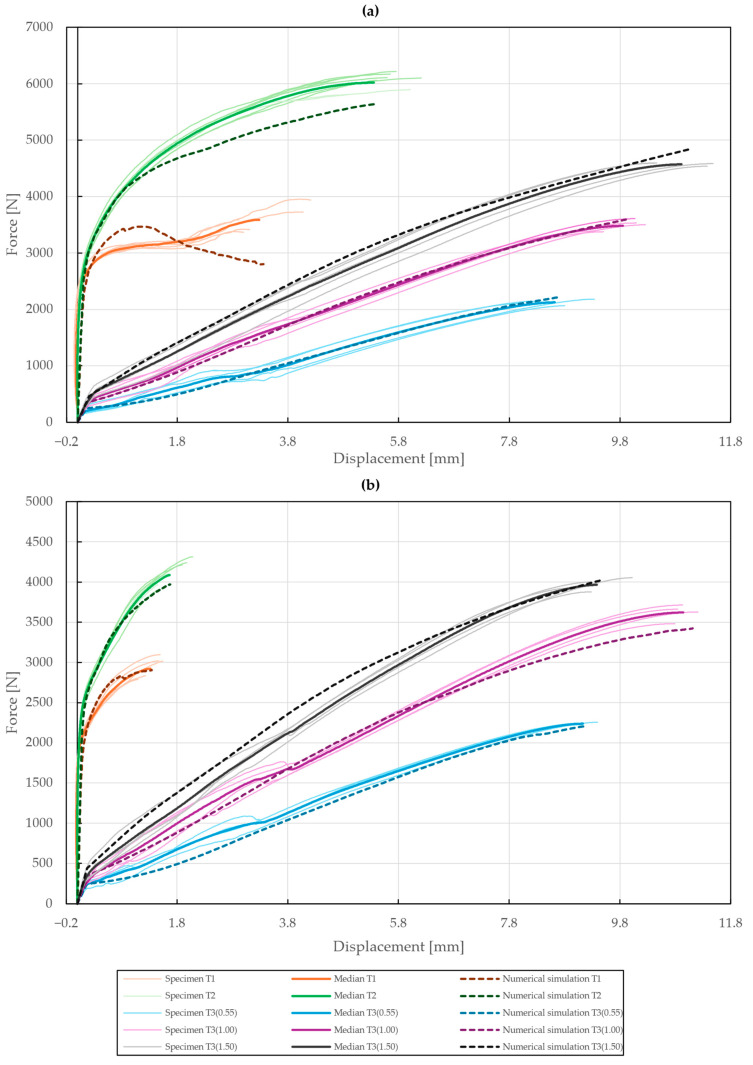
Force–displacement curve of single-lap shear (T1), double-lap shear (T2), and pure normal loading (T3) of specimens riveted with DIN 7337 blind rivets and the corresponding results of their fitted non-linear numerical simulations: (**a**) stainless steel blind rivet DIN 7337—4.8 × 8 A2/A2; (**b**) galvanized carbon steel blind rivet DIN 7337—4.8 × 8 St/St.

Using this methodology, a substantial agreement between the non-linear numerical simulation and experimental results was achieved with a cumulative absolute difference of 7.47% ($\bar{∆}=1.14$), proving that correct assumptions and simplifications to the FE models were made despite the sheer amount of variables. Additionally, an increase in computational efficiency was observed, allowing for the fitting of six different non-linear numerical simulations based on two subsequent optimizations. The comprehensive results of the rivet joint calibration are shown in Figure 30. The results of deformations and the observed failure modes conform to the experimental data and thus a very precise representation of the rivet joints’ behavior when subjected to shear and normal loading was created. Therefore, the selected material model, consisting of isotropic elasticity and Chaboche kinematic hardening, was proven to be appropriate for accurate description and further assessment of blind rivet joint behavior. Additionally, the use of optiSLang allowed for the statistical analysis of the performed calibration through the determination of the most important parameters.

## 7. Conclusions

The performance of blind rivet joints in galvanized steel sheets under three distinct loading conditions—single-lap shear, double-lap shear, and pure normal tensile loading—was examined. Using both experimental testing and finite element analysis (FEA), we assessed how different rivet materials—stainless steel (A2/A2) and galvanized carbon steel (St/St)—influence the joints’ behavior. Based on the results, a methodology was proposed for the fitting of multiple non-linear numerical simulations to the experimental results through the calibration of the material parameters of the rivets and adherends, providing a solid basis for the FE modeling of the rivet joint while accurately assessing its behavior.

The normal load tests focused on tensile forces perpendicular to the joint, highlighting the influence of sheet thickness on joint performance. In contrast, the shear tests evaluated how joints withstand forces parallel to the sheet plane. Rivet joints in the single-lap shear configuration experienced additional bending stress due to misalignment, i.e., a tearing out of the rivet under tensile loading, reducing their load-bearing capacity compared to double-lap shear joints. The double-lap shear configuration eliminated this bending effect, resulting in higher strength and more consistent failure modes.

Across all tests, stainless steel rivets consistently outperformed galvanized carbon steel rivets, while different failure modes were observed. The A2/A2 rivets demonstrated higher load-bearing capacities and greater deformation tolerance, making them more suitable for applications requiring enhanced strength and durability.

It was found that the performance of blind rivet joints is significantly affected by both the loading conditions and the materials used. Double-lap shear joints offer superior strength due to their better stress distribution, while single-lap shear joints are more susceptible to bending-induced stress concentrations. Under normal tensile loads, increasing the sheet thickness can improve joint performance, particularly when using stainless steel rivets.

The possibilities of simplifying the rivet joint using 1D and 2D elements, such as CBEAM and CFAST, will be investigated in future work, which will aim to provide a comprehensive guide to modeling rivet joints—from sub-models to large-scale FE models subjected to complex multi-axial loading—while maintaining this overall accuracy and computational efficiency. To specify, a comprehensive knowledge base could be created based on the extensive data obtained in this research paper. Then, with the use of modern computational methods, such as artificial intelligence and digital twins, a more efficient and precise assessment of real structures utilizing rivet joints, e.g., truss structures, bridges, or parts of railway infrastructure, will be made possible. Finally, the database could be further expanded and implemented into PLM systems to help streamline the FEA by providing accurate, experimentally proven material models of various rivets, along with their simplified versions for rapid virtual prototyping in pre-production phases.

To further advance the research of rivet joint behavior and its applicability in practical engineering, the coupling of various complex loading conditions, such as cyclic fatigue, corrosion, and temperature variations should be considered. This could be carried out using Multiphysics FE simulations, which are commonly used to couple the thermal and structural loading of a structure.

## Figures and Tables

**Table 1:** Chemical composition of DX51D + Z275 steel.

C [%]	Mn [%]	Si [%]	P [%]	S [%]	Cu [%]	Cr [%]	Ni [%]	Al [%]	V [%]	Mo [%]
0.04	0.24	0.023	0.007	0.004	0.04	0.03	0.023	0.03	0.001	0.002

**Table 2:** Mechanical properties of DX51D + Z275 steel.

Mechanical Property	[Unit]	DX51D + Z275
Test temperature	[°C]	23
Average coating thickness	[g/m^2^]	286
$\mathrm{Yield}\;\mathrm{strength}\;R_{eL}$	[MPa]	315
$\mathrm{Tensile}\;\mathrm{strength}\;R_{m}$	[MPa]	367
$\mathrm{Total}\;\mathrm{elongation}\;A_{80}$	[%]	39.6

**Table 3:** Mechanical properties of DIN 7337—4.8 × 8 blind rivets.

Mechanical Property	[Unit]	DIN 7337—4.8 × 8	
		A2/A2	St/St
Maximum normal load F	[N]	5000 ^1^	3700 ^1^
Maximum shear load V	[N]	4000 ^1^	2900 ^1^
$\mathrm{Maximum}\;\mathrm{normal}\;\mathrm{stress}\;\sigma_{max}$	[MPa]	442.1	302
$\mathrm{Maximum}\;\mathrm{normal}\;\mathrm{stress}\;\sigma_{max}$	[MPa]	288.7	188.4

^1^ The values for the maximum normal and shear load were provided by the rivet’s manufacturer.

**Table 4:** Overview of failure modes observed in riveted specimens.

Specimen Configuration	Failure Mode	
	DIN 7337—4.8 × 8 A2/A2	DIN 7337—4.8 × 8 St/St
Single-lap shear T1	Rivet slippage	Rivet failure
Double-lap shear T2	Steel sheet failure	Rivet failure
Pure normal T3 (t=0.55 mm)	Rivet slippage	Rivet slippage
Pure normal T3 (t=1.00 mm)	Rivet slippage	Rivet slippage
Pure normal T3 (t=1.50 mm)	Rivet slippage	Rivet failure

**Table 5:** Calibrated material parameters of DX51D + Z275 steel sheet specimen.

Material Parameter	[Unit]	DX51D + Z275
Young’s modulus E	[MPa]	$2.4794\times 10^{5}$
Poisson’s ratio μ	[–]	0.29029
$\mathrm{Yield}\;\mathrm{stress}\;R_{e}$	[MPa]	258.96
$\mathrm{Material}\;\mathrm{constant}\;C_{1}$	[MPa]	2442.3
$\mathrm{Material}\;\mathrm{constant}\;\gamma_{1}$	[–]	10.831

**Table 6:** Calibrated material parameters of DIN 7337—4.8 × 8 blind rivets.

Material Parameter	[Unit]	DIN 7337—4.8 × 8	
		A2/A2	St/St
Young’s modulus E	[MPa]	$2.7021\times 10^{5}$	$2.9694\times 10^{5}$
Poisson’s ratio μ	[–]	0.27672	0.33747
$\mathrm{Yield}\;\mathrm{stress}\;R_{e}$	[MPa]	425.93	291.13
$\mathrm{Material}\;\mathrm{constant}\;C_{1}$	[MPa]	4784.4	2980.6
$\mathrm{Material}\;\mathrm{constant}\;\gamma_{1}$	[–]	3.6501	17.724

## Data Availability

The original contributions presented in the study are included in the article, further inquiries can be directed to the corresponding authors.
